# *In vivo* ossification of a scaffold combining β-tricalcium phosphate and platelet-rich plasma

**DOI:** 10.3892/etm.2014.1969

**Published:** 2014-09-15

**Authors:** DA ZHONG, CHENG-GONG WANG, KE YIN, QIANDE LIAO, XING ZHOU, AN-SONG LIU, LING-YU KONG

**Affiliations:** Department of Orthopedics, Xiangya Hospital, Central South University, Changsha, Hunan 410008, P.R. China

**Keywords:** tricalcium phosphate, platelet-rich plasma, tissue engineering, bone defect, bone marrow stromal cells

## Abstract

Tricalcium phosphate (TCP) and platelet-rich plasma (PRP) are commonly used in bone tissue engineering. The aim of the present study was to investigate a composite that combined TCP with PRP and assess its effectiveness in the treatment of bone defects. Cavity-shaped bone defects were established on the tibiae of 27 beagle dogs, and were repaired by pure β-TCP with bone marrow stromal cells (BMSCs), β-TCP/PRP with BMSCs and autogenic ilium. The samples were harvested at 4, 8 and 12 weeks, and bone regeneration was evaluated using X-ray radiography, immunocytochemical staining of osteocalcin (OCN), hematoxylin and eosin staining and reverse transcription-polymerase chain reaction analyses. Biomechanical tests of the scaffolds were performed at the 12th week after scaffold implantation. When using pure β-TCP as a scaffold, the scaffold-bone interface was clear and no material adsorption and bone healing was observed. Substantial bone regeneration was observed when the tibial defects were restored using β-TCP/PRP and autogenic ilium. Furthermore, the mRNA expression levels of OCN, alkaline phosphatase and collagen type I α1 were significantly higher in the animals with β-TCP/PRP scaffolds at 8 and 12 weeks following implantation compared with those in the animals with the pure β-TCP scaffolds. The maximum load and compressive strength of the β-TCP/PRP scaffolds were similar to those of the autogenic ilium; however, they were significantly higher than those of the pure β-TCP scaffold. Thus, the β-TCP/PRP composite may be used as a potential scaffold to carry *in vitro* cultured BMSCs to treat bone defects.

## Introduction

The rapid development in the field of tissue engineering has resulted in the existence of alternatives for the treatment of large segmental bone defects. In general, tissue-engineered bone is composed of seed cells, a scaffold and growth factors. At present, mesenchymal stem cells are preferentially used as seed cells due to their wide range of sources, the low degree of damage they inflict on the body and their capacity for multi-directional differentiation into osteoblasts, neurocytes, chondrocytes and epithelial cells through induction with certain conditioned media (1,2). The scaffold determines the structure and mechanical properties of the tissue engineered-bone. Previous studies have revealed that β-tricalcium phosphate (β-TCP), a common scaffold material with effective biocompatibility, histocompatibility and mechanical properties, could supply the seed cells with an appropriate environment for *in vitro* culture (3–5).

Intensive study in the field of bone tissue engineering has focused on investigating growth factors, since they potentially promote the proliferation and differentiation of seed cells, as well as creeping substitution and the formation of new bones. It has been indicated that there is a high concentration of numerous growth factors in platelet-rich plasma (PRP), including platelet-derived growth factor (PDGF), transforming growth factor (TGF)-β1, TGF-β2, insulin-like growth factor (IGF), vascular endothelial growth factor (VEGF), epidermal growth factor (EGF) and endothelial cell growth factor (6). These growth factors promote the proliferation, migration and differentiation of seed cells, as well as facilitating collagen protein synthesis and vascularization (7,8). Furthermore, previous studies investigating the potential application of PRP in bone tissue engineering have revealed that PRP may enhance osteogenesis and accelerate bone defect healing to various degrees (9–11). However, others studies investigating PRP have disputed these applications (12,13). These studies argue that the composition of PRP is so complicated that it is difficult to clearly distinguish the single effect of each growth factor. In 2006, van den Dolder *et al* (14) quantitatively analyzed the concentrations of different growth factors, including TGF-β1, TGF-β2, PDGF-AA, PDGF-AB and PDGF-BB, in rat, goat and human PRP. They also investigated the effects of PRP on cell growth and differentiation by culturing rat bone marrow cells in PRP-coated wells for 16 days in osteogenic media. Although the results demonstrated that all three types of PRP stimulated initial cell growth due to the presence of osteoinductive growth factors, the data could not be generalized due to large interspecies variations. In the study by Tajima *et al* (15) bone marrow stromal cells (BMSCs) were suspended in PRP or platelet-poor plasma (PPP), which were subsequently introduced into porous β-TCP blocks and implanted into subcutaneous sites in rats. The results revealed that the implants prepared using PPP had a greater osteoinductive capability compared with those prepared with PRP.

A literature review revealed that PRP has not been commonly studied *in vivo* for the construction of tissue-engineered bone, particularly for the treatment of bone defects in medium or large animal models (16,17). In the present study, the osteogenic characteristics of a scaffold combining β-TCP and PRP were investigated *in vivo* by establishing a proximal tibial bone defect model in beagle dogs, with mesenchymal stem cells used as seed cells. The present study provides a useful foundation for the further study of bone tissue engineering in medium or large animals.

## Materials and methods

### Isolation, cultivation, purification and proliferation of beagle BMSCs

The procedures carried out in the present study were approved by the Ethics Committee of Xiangya Hospital Affiliated to Central South University (Changsha, China). An adult beagle dog (12 months old and weighing 10 kg) was obtained from the Animal Department of Xiangya Medical College (Changsha, China). The dog was intravenously anesthetized with 3% pentobarbital (1.0 ml/kg; Propbs Chemical & Pharmaceutical Co. Ltd., Beijing, China) and 3 ml bone marrow was extracted using sterile techniques. The marrow was washed and diluted in phosphate-buffered saline (PBS) solution and poured slowly over Percoll (1.079 g/ml; GE, Shanghai, China) with a volume of 1:1. The mixture was centrifuged at 223.6 × g for 15 min, following which the liquid formed four layers. The second layer, which was white and membrane-like, was carefully drawn, washed in PBS solution and centrifuged at 55.9 × g for 5 min. Following removal of the supernatant, Dulbecco’s modified Eagle’s medium - Low Glucose (DMEM-LG; Gibco^®^; Invitrogen Life Technologies, Carlsbad, CA, USA) combined with 10% fetal calf serum (Shanghai DingGuo Biotech Co. Ltd., Shanghai, China), containing 100 μg/ml penicillin and 100 μg/ml streptomycin, was added to resuspend the cells. The cells were seeded in a 25-cm^2^ culture flask at a density of 2×10^5^ cells/cm^2^ and cultured in a CO_2_ incubator (Queue^®^, Asheville, NC, USA) following the addition of 4 ml DMEM-LG with fetal calf serum. The media was changed after the first 48 h and every three days thereafter. Finally, 0.25% trypsin (Sigma-Aldrich, St. Louis, MO, USA) digestion was performed when cell fusion was observed in 80–90% of the anchorage-dependent cells. Serial subculture was performed at a proportion of 1:3.

### Preparation of PRP

Venous blood (10 ml) was collected from the hind limb of the beagle and placed in a 15-ml centrifuge tube containing 1 mg sodium citrate. PRP was extracted using the two-step centrifugation method. Firstly, the blood sample was centrifuged at 125.775 × g for 10 min. The upper plasma layer and the erythrocytes within 2 mm of the interface were transferred to another centrifuge tube. The sample was then centrifuged at 724.464 × g for 10 min and the upper plasma layer that contained a small amount of suspending platelets was removed. The remaining plasma (~1 ml) was PRP. PRP was prepared using this method and stored at −70°C until required.

### Preparation of the β-TCP and PRP composite (β-TCP/PRP)

The β-TCP samples (cylindrical; diameter, 1.0 cm; height, 4.0 cm) were cut into 0.5-cm slices using a clean bench (GS-15R; Haier, Qingdao, China). The slices were repeatedly washed with saline and sterilized at 130°C following drying. The slices were immersed in PRP until the TCP material was completely saturated by the plasma. The activating agent (10% calcium chloride solution containing 100 μg/ml bovine thrombin) was added at a ratio of 1:1, and the mixture was reacted in a 37°C water bath to form the β-TCP/PRP gel composite.

### Preparation of the scaffold with the seeding of BMSCs onto the composites

Third-generation BMSCs were collected and the cell suspension density was adjusted to 1.0×10^5^/l. The cell suspension was implanted onto the β-TCP and β-TCP/PRP composites using a micropipette (50 μl for each bracket). The materials were stored for 4 h in the CO_2_ incubator (37°C and 5% CO_2_ saturated humidity) to allow further adhesion of the cells. A total of 2 ml high-glucose DMEM (Gibco; Invitrogen Life Technologies) containing 10% fetal calf serum, dexamethasone (1×10^−7^ M), β-glycerophosphate (10 mM) and ascorbic acid (500 mg/l) was added per well. Finally, the composite was placed in the CO_2_ culture incubator for a further week.

### Establishment of the bone defect in the upper segment of the tibia in beagles

A total of 27 beagle dogs were intravenously anesthetized with 3% pentobarbital (1.0 ml/kg; Propbs Chemical & Pharmaceutical Co. Ltd.). Bilateral incisions were made at the medial lower extremities and the medial tibiae were accessed following periosteal stripping. Cavity-shaped bone defects (<10 mm in diameter) were established on both sides at 2 cm below the medial tibial plateau.

The dogs were classified into three groups (n=9 per group) and implanted with different types of scaffolds. The β-TCP scaffold with seeded BMSCs was implanted into Group I animals and the β-TCP/PRP scaffold with seeded BMSCs was implanted into Group II animals. Autogenic ilium was used for Group III animals. Following the implantation of the scaffolds, the periosteum in the surgical area was removed and the subcutaneous tissue and skin were tightly sutured. The dogs were administered penicillin to prevent postoperative infection.

### Evaluation methods

The beagles in each group were examined using X-ray radiographs at three time-points: 4, 8 and 12 weeks after implantation (with three radiographs at each time-point). At each time-point, three dogs were sacrificed from each group by an intravenous injection of 3% pentobarbital (1.0 ml/kg), followed by an intravenous injection of air into the hind limb. Subsequently, immunocytochemical staining of osteocalcin (OCN), hematoxylin and eosin (H&E) staining, reverse transcription-polymerase chain reaction (RT-PCR) analysis and biomechanical tests were performed in order to examine callus formation, the regeneration of bone defects and the reaction of the surrounding tissue.

#### Immunocytochemical staining of OCN

Sections (3–5 μm) were dewaxed and hydrated, washed with PBS solution and incubated with 3% H_2_O_2_ for 5–10 min to eliminate the endogenous peroxidase activity. The sections were subsequently washed in distilled water and immersed in PBS solution for 5 min. Mouse anti-dog OCN polyclonal-antibody (dilution 1:100; Abcam, Cambridge, MA, USA) was added and the sections were incubated overnight at 4°C. The sections were then washed in PBS solution (three times, 5 min each), and biotinylated anti-mouse immunoglobulin G secondary antibody (Zhongshan Jinqiao Co. Ltd., Beijing, China) was added followed by incubation at 37°C for 10–15 min. Following washing with PBS solution (three times, 5 min each), horseradish peroxidase-labeled streptavidin (Zhongshan Jinqiao Co. Ltd.) was added and the sections were incubated at 37°C for 10–15 min. The sections were then washed in PBS solution (three times, 5 min each) and 3,3′-diaminobenzidine tetrahydrochloride chromogenic agent (Zhongshan Jinqiao Co. Ltd.) was added for color development. Following washing in distilled water, the sections were counterstained with hematoxylin and mounted. OCN was observed using an optical microscope (CX21; Olympus, Tokyo, Japan).

#### H&E staining

Following dewaxing and hydration, the sections were soaked in hematoxylin (Weigert iron hematoxylin stain; Shanghai Harling Biotechnology Co. Ltd, Shanghai, China) for 5 min, washed with distilled water and differentiated with 1% hydrochloric acid alcohol for 30 sec. Subsequent to washing again in distilled water, the sections were immersed in eosin for 2 min, washed with distilled water and dehydrated by a graded ethanol series. Finally, the sections were cleaned with xylol, mounted in neutral resin and observed using optical microscopy.

#### RT-PCR

The sequences of the primers used were as follows: OCN forward, GAATCCCGCAAAGGTGGCTGA CCACATTGGCTT and reverse, AAGCCAATGTGGTCA GCCACCTTTGCGGGATTC (185 bp); alkaline phosphatase (ALP) forward, GAGTGACACGGACAAGAAGCC CCAACCAGGACCACTGTGCCTCA and reverse, TGA GGCACAGTGGTCCTGGTTGGGGCTTCTTGTCCGTGT CACTC (292 bp); collagen type I α1 (CollA1) forward, TCCAGGGTTCCAACGAGAAGACCTCCCGTTTGCC and reverse, GGCAAACGGGAGGTCTTCTCGTTGGAA CCCTGGA (145 bp); and GAPDH forward, AAGGTCGGA GTCAACGGATTTGGCATCAGCAGAAGGAGCAG and reverse, CTGCTCCTTCTGCTGATGCCAAATCCGTTG ACTCCGACCTT (372 bp). The sequences were designed using Primer Premier software (Premier Biosoft, Palo Alto CA, USA). The total RNA was extracted from each sample using a TRIzol^®^ kit (Molecular Research Centre, Inc., Cincinnati, OH, USA). The cDNA was subsequently synthesized using a RT kit (R0901-100ML; Sigma-Aldrich). Firstly, the total RNA (2.0 μl), Oligo(dT)18 primer (1.0 μl) and DNase/RNase-free ddH_2_O (9.0 μl) were added to a 0.2-ml RNase-free Eppendorf tube. Following mixing and mild centrifugation (13.975 × g for 90 sec at 22–24°C), the tube was reacted at 70°C for 5 min. The 5× RT buffer (4.0 μl), 10 mM deoxynucleotide triphosphates (dNTPs; 2.0 μl) and RNasin^®^ (1.0 μl) were added to the tube and mild centrifugation was carried out following mixing. The solution was reacted at 37°C for 5 min, and 1.00 μl M-MLV reverse transcriptase (M1302-40KU; Sigma-Aldrich) was added. The mixture was reacted at 42°C for 60 min and then at 70°C for 10 min. The synthesized cDNA was stored at −20°C until required. PCR was subsequently performed. Firstly, 10× PCR buffer (5.0 μl), 10 mM dNTPs (2.0 μl), MgCl_2_ (3.0 μl), 10 pmol/μl target gene forward primer (1.6 μl), 10 pmol/μl target gene reverse primer (1.6 μl), 10 pmol/μl β-actin gene forward primer (1.6 μl), 10 pmol/μl β-actin gene reverse primer (1.6 μl), cDNA (3.0 μl), 1.0 U/μl *Taq* DNA polymerase (2.6 μl) and DNase/RNase-free ddH_2_O (28.0 μl) were added to a 0.2-ml PCR tube. The mixture was subsequently transferred to an Eppendorf tube and placed into the TripleMaster PCR system (Eppendorf, Hamburg, Germany) for DNA synthesis according to the designed primers (OCN, ALP and CollA1). The PCR products were visualized on an ethidium bromide-stained agarose gel.

#### Biomechanical tests

The tibiae of three dogs from Groups I, II and III were obtained 12 weeks after surgery. The meniscus and attached ligaments and muscle tissues were carefully dissected. The ends of the tibiae were closed with denture powder and polished with fine sandpaper to create a smooth surface. The tibiae were placed vertically in a universal material tester (Instron 8032; TestResources, Shakopee, MN, USA) for compression analyses with a loading speed of 5 mm/min. The maximum load (the maximum external force that the tibia could bear) and the maximum compressive strength (maximum load/contact area) were recorded during the experiment.

### Statistical analysis

Data are presented as the mean ± standard deviation. Statistical analyses were performed using SPSS 12.0 software (SPSS, Inc., Chicago, IL, USA). A two-sample Student’s t-test was used to evaluate the differences between groups. Differences were considered statistically significant with a two-tailed value of P<0.05.

## Results

### Observation of the implanted scaffolds in the beagle dogs in each group

**Figure 1:**
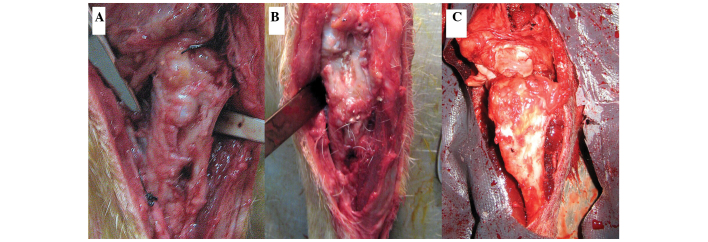
Observation of the implanted scaffolds in the dogs in Groups (A) I (pure β-TCP), (B) II (β-TCP/platelet-rich plasma) and (C) III (autogenic ilium). No systemic or local inflammation or toxicity was detected in any group. β-TCP, β-tricalcium phosphate.

All wounds healed well following implantation of the scaffolds into the tibial defects of the dogs in each group. No systemic or local inflammation or toxicity were observed in any group. No significant inflammation or rejection was detected for any of the implanted scaffolds, as shown in Fig. 1.

### Postoperative X-ray examination

**Figure 2:**
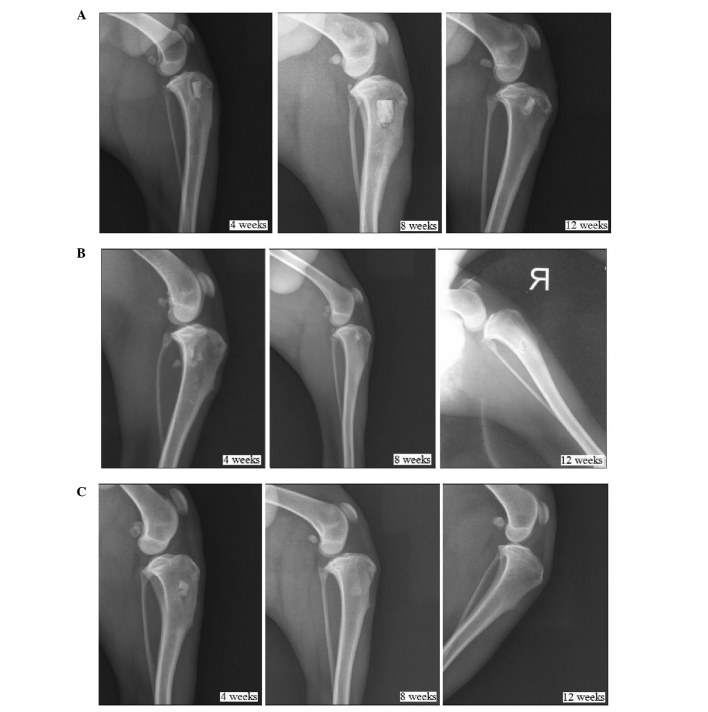
X-ray radiographs showing bone defect healing at the different time-points (4, 8 and 12 weeks) after implantation of the scaffolds. (A) Group I (pure β-TCP). No material absorption or bone healing was observed until the 12th week following implantation. (B) Group II (β-TCP/platelet-rich plasma). The scaffold-bone interface was indistinct and bone healing was generally complete at the 12th week following implantation. (C) Group III (autogenic ilium). Indistinct fusion of the scaffold-bone interface was detected at the 4th week and complete bone healing was observed at the 12th week following implantation. β-TCP, β-tricalcium phosphate.

No material absorption or bone healing was observed in Group I (implantation of β-TCP with seeding of BMSCs) until 12 weeks after implantation. The scaffold-bone interface remained clear. The shadow around the scaffold suggested the occurrence of bone resorption, as shown in Fig. 2A. In Group II (implantation of β-TCP/PRP with seeding of BMSCs), the scaffold-bone interface was indistinct, which was indicative of a relatively fast fusion. Bone healing was generally complete 12 weeks after the implantation. However, as shown in Fig. 2B, a small amount of high-density shadow remained, which may have been the remaining TCP. The most rapid bone healing was observed in the animals in Group III (implantation of autogenic ilium). Indistinct fusion of the scaffold-bone interface was detected just 4 weeks after the implantation, and complete bone healing was observed at 12 weeks after the implantation (Fig. 2C).

### Immunohistochemical staining of OCN and H&E staining

**Figure 3:**
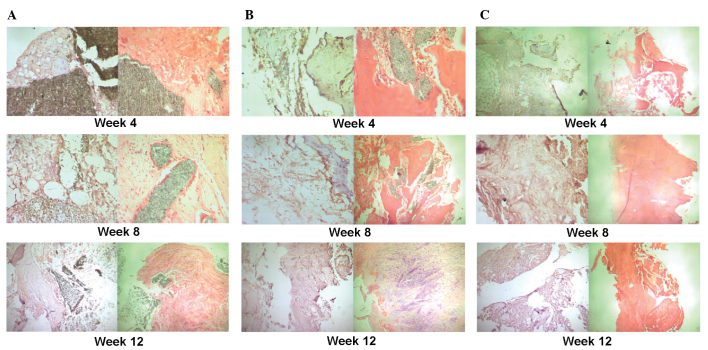
Immunohistochemical staining of osteocalcin and hematoxylin and eosin staining at different time-points (4, 8 and 12 weeks) following implantation of the scaffolds. (A) Group I (pure β-TCP). At 4 weeks following implantation, new formation of cartilage and bone was observed at the edges of the scaffold (magnification, ×200). The expression of newly formed cartilage and bone increased at 8 weeks following implanation and a number of osteogenic cells arranged in a spiral were observed (magnification, ×200). At 12 weeks following implantation there was an even higher expression of newly formed cartilage and bone at the edges of the scaffold cells and the osteogenic cells were arranged in a spiral; however, the majority of the β-TCP remained detectable (magnification, ×100). (B) Group II (β-TCP/PRP). At 4 weeks following implantation, new formation of cartilage and bone was observed at the edges of the scaffold and the scaffold began to degrade (magnification, ×200). The expression of newly formed cartilage and bone increased at 8 weeks following implanation and the scaffold was further degraded and absorbed (magnification, ×100). This continued at 12 weeks following impantation where the formation of capillary vessels was also detected (magnification, ×200). (C) Group III (autogenic ilium). At 4 weeks following implantation, the new formation of cartilage, bone and capillary vessels was observed. This increased at 8 and 12 weeks following implantation with much mature bone tissue observed at 12 weeks following implantation (magnification, ×100).

The results obtained from the immunohistochemical staining of OCN and H&E staining were similar among the three groups. New cartilage formation was observed in each group, with higher expression in the samples from Groups II and III compared with those from Group I (Fig. 3).

### RT-PCR

**Figure 4:**
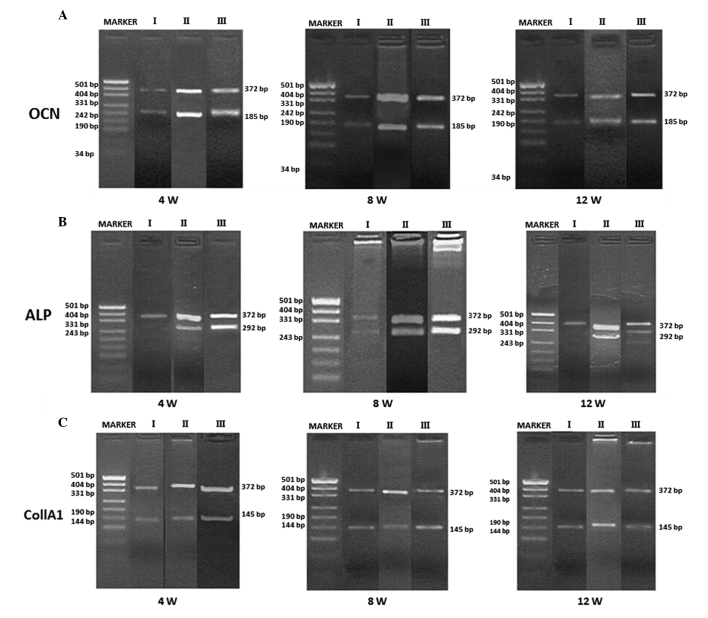
Expression levels of (A) OCN, (B) ALP and (C) Col1A1 as detected by reverse transcription-polymerase chain reaction at the three time-points (4, 8 and 12 weeks) following implantation of the scaffolds for Groups I (pure β-TCP), II (β-TCP/platelet-rich plasma) and III (autogenic ilium). OCN, osteocalcin; ALP, alkaline phosphatase; CollA1, collagen type I α1; β-TCP, β-tricalcium phosphate.

Using GAPDH as an internal control (expressed as a 372-bp band), the expression levels of OCN, ALP and Col1A1 were detected using RT-PCR at the three time-points (4, 8 and 12 weeks after implantation) for the samples in each group. OCN, ALP, and Col1A1 were weakly expressed in all groups 4 weeks after implantation. At 8 and 12 weeks after implantation, the expression levels of OCN, ALP, and Col1A1 in Groups II and III were higher than those in Group I, but all were strongly positive (Fig. 4). According to the comparisons between the target bands and the reference gray values, the expression levels of OCN, ALP and Col1A1 were not statistically different at 4 weeks after implantation. At 8 and 12 weeks following implantation, the expression level of each mRNA in Groups II and III was statistically higher than that of the same mRNA in Group I (P<0.05). However, no significant difference was identified between the expression levels of the mRNAs in Groups II and III (P>0.05), as shown in Table I.

### Biomechanical tests

Table II shows the maximum load and compressive strength of the tibia segments in each group, as measured 12 weeks after implantation. The maximum load and compressive strength for the samples in Groups II and III were significantly than those for the samples in Group I (P<0.05). However, no statistical differences were identified in these values between Groups II and III (P>0.05).

## Discussion

Seed cells, scaffolds and growth factors play important roles in bone tissue engineering. Numerous studies have focused on the functional mechanism of PRP in bone tissue engineering. However, few studies have explored the osteogenic characteristics of PRP in medium and large animal models for the treatment of bone defects (18–20). In the present study, a β-TCP and PRP gel composite was introduced as the scaffold to carry BMSCs, making it simultaneously osteoconductive and osteoninductive. The scaffolds were implanted into the tibial bone defects of beagle dogs following one week of *in vitro* directional cell osteogenic induction. Compared with the autogenic ilium and pure β-TCP scaffolds with BMSC seeding, the β-TCP/PRP scaffold exhibited no significant inflammation or rejection, with excellent histocompatibility. As observed from the X-ray radiograph images captured at 8 and 12 weeks after the implantation, the osteogenic effects of the β-TCP/PRP scaffolds with BMSC seeding were superior to those of the pure β-TCP scaffolds, and were similar to those of the autogenic ilium. General bone healing was observed 12 weeks after the implantation of the β-TCP/PRP scaffolds, indicating their effective osteogenic capability.

The most common method of promoting the differentiation of BMSCs into osteoblasts is chemical drug induction (21–23). In the present study, a specific inducing media, which included dexamethasone, β-glycerophosphate and ascorbic acid, was used to facilitate the *in vitro* differentiation of BMSCs into osteoblasts. Dexamethasone promotes osteoblast differentiation, is able to regulate the secretion of IGF and facilitates extracellular matrix collagen synthesis. β-glycerophosphate can supply phosphate ions to osteoblasts, thereby promoting the deposition and calcification of physiological calcium, which is required for the formation of mineralized nodules. Ascorbic acid can adjust the activity of ALP and the synthesis of non-collagen matrix proteins. PRP contains a high concentration of growth factors, which promote seed cell proliferation, movement, differentiation, collagen synthesis and vascularization. Marx *et al* (24) proposed that the reason that PRP is beneficial for bone defect healing may be attributed to the high concentration of PDGF and TGF-β in PRP. Fennis *et al* (25) found that the addition of PRP to an autogenic bone graft had a positive effect on bone healing in the early stages. Cell differentiation increased as the direct effect of PRP declined and further proliferation and differentiation promoted the healing of bone defects at a high level. The high concentrations of PDGF and TGF-β work effectively in the early stages of bone defect healing by increasing the number of BMSCs in the trauma area along with other growth factors in the PRP, such as basic fibroblast growth factor (24,25). Vascularization of the implanted tissue-engineered bone usually occurs two weeks after implantation (26). The growth factors in PRP, including VEGF, IGF, EGF and PDGF, are able to promote revascularization of the scaffold (6). Good revascularization accelerates local blood supply in the bone defect areas and further promotes osteogenesis(26).

β-TCP is a type of bioactive ceramic, which is degraded *in vivo* by dissolving and by phagocytic cells. Since its degradation products are non-toxic, non-teratogenic and non-tumorigenic, β-TCP is considered a promising material for bone tissue engineering (27). An ideal scaffold requires a degradation time that is in accordance with osteoblast growth and proliferation, as well as matrix secretion. The present study demonstrated, using immunohistochemical staining of OCN and H&E staining, that the calcium phosphate in the β-TCP/PRP scaffold implanted into the beagles generally degraded 12 weeks after implantation, whereas the majority of the calcium phosphate remained in Group I (implantation of β-TCP with BMSC seeding). Furthermore, OCN expression was stronger in the β-TCP/PRP group compared with that in the TCP group at all time-points, with an increased formation of new cartilage. The results suggest that the degradation rate of the β-TCP/PRP scaffold matched the proliferation rate of the BMSCs and the synthesis and secretion of the matrix. Therefore, the β-TCP/PRP scaffold is suitable to be applied in bone tissue engineering to treat bone defects. The tissue sections were prepared following the sacrifice of the dogs at 4, 8 and 12 weeks after implantation. The statistical analysis of the mRNA expression levels for OCN, ALP and Col1A1 further indicated that the β-TCP/PRP scaffolds were similar to autogenous iliac bone in the promotion of osteogenesis, but were significantly more effective than the pure β-TCP scaffolds. These results corresponded with those obtained from the X-ray radiographs and immunohistochemical and H&E stains.

The mechanical properties of a scaffold are closely associated with its degradation rate. The initial mechanical strength should be able to resist physiological stress so that it does not experience collapse during the growth of tissue cells. Since mechanical strength may also influence the tension generated by the intracellular skeleton, a resilient surface provides a good environment for fiber configuration, as well as cell expansion and differentiation. The β-TCP/PRP scaffold has superior mechanical properties and degradation rates compared with cancellous bone. In the present study, the tibial specimens collected 12 weeks after implantation were placed in the Instron universal material tester for vertical compression experiments. The results revealed that the β-TCP/PRP scaffold had similar mechanical properties to iliac bone, but superior mechanical properties to the pure β-TCP scaffold.

In the present study, the tibial bone defects in beagles were successfully repaired when a β-TCP/PRP gel composite was used as the scaffold carrying *in vitro*-cultured BMSCs. The osteogenic effect of the of β-TCP/PRP scaffold was similar to that of the autogenic ilium. As a novel application in bone tissue engineering, PRP is likely to promote new bone formation through the release of the growth factors it contains. Thus, β-TCP/PRP may be an ideal scaffold to treat bone defects with tissue engineering.

## Figures and Tables

**Table I:** mRNA expression levels of OCN, ALP and Col1A1 for Groups I, II and III at different time-points following implantation of the scaffolds.

Group	Time-point (weeks)	No. samples	OCN/GAPDH	Col1A1/GAPDH	ALP/GAPDH
I	4	3	0.82±0.09	0.74±0.10	1.11±0.77
	8	3	0.85±0.12	0.93±0.10	0.99±0.11
	12	3	0.89±0.07	0.89±0.07	0.80±0.42
II	4	3	0.86±0.11	0.76±0.10	1.14±0.10
	8	3	1.01±0.13	1.15±0.08	1.12±0.10
	12	3	1.12±0.11	1.13±0.10	0.96±0.35
III	4	3	0.85±0.10	0.77±0.07	1.20±0.09
	8	3	1.08±0.09	1.19±0.10	1.15±0.10
	12	3	1.12±0.11	1.11±0.11	0.95±0.59

Data are presented as the mean ± standard deviation. Group I, pure β-TCP; Group II, β-TCP/platelet-rich plasma; Group III, autogenic ilium. β-TCP, β-tricalcium phosphate; OCN, osteocalcin; CollA1, collagen type I α1; ALP, alkaline phosphatase.

**Table II:** Maximum load and compressive strength of the tibia segments for Groups I, II and III at 12 weeks following implantation of the scaffolds.

Group	No. samples	Maximum load (N)	Maximum compressive strength (MPa)
I	6	3637.5±129.6	17.5±0.6
II	6	3917.7±96.8	20.2±0.8
III	6	3946.7±106.2	19.6±0.8

Data are presented as the mean ± standard deviation. Group I, pure β-TCP; Group II, β-TCP/platelet-rich plasma; Group III, autogenic ilium. β-TCP, β-tricalcium phosphate.
